# Supportive Accountability and Mobile App Use in a Tobacco Control Intervention Targeting Low-Income Minority Mothers Who Smoke: Observational Study

**DOI:** 10.2196/28175

**Published:** 2021-07-02

**Authors:** Stephen J Lepore, Bradley N Collins, Howard W Killam, Barbara Barry

**Affiliations:** 1 Department of Social and Behavioral Sciences Temple University Philadelphia, PA United States; 2 User Centered Design Inc Ashburn, VA United States

**Keywords:** tobacco cessation, smoking, mHealth, mobile apps, smartphone, mobile phone, adherence, engagement, minority health

## Abstract

**Background:**

Smartphone mobile apps are frequently used in standalone or multimodal smoking cessation interventions. However, factors that impede or improve app usage are poorly understood.

**Objective:**

This study used the supportive accountability model to investigate factors that influence app usage in the context of a trial designed to reduce maternal smoking in low-income and predominantly minority communities.

**Methods:**

We conducted a secondary analysis of data (N=181) from a randomized controlled trial that included a smoking cessation app (QuitPal-m). Supportive accountability was measured by the number of times a participant was advised by their cessation counselor to use QuitPal-m. Participants reported app use helpfulness and barriers. Investigators tracked reported phone and technical problems that impeded app use.

**Results:**

Most participants rated the app as very helpful (103/155, 66.5%), but daily use declined rapidly over time. App use was positively related to the level of perceived app helpfulness (*P*=.02) and education (*P*=.002) and inversely related to perceived barriers (*P*=.003), phone technical problems (*P*<.001), and cigarettes smoked per day at the end of treatment (*P*<.001). Participants used the app a greater proportion of the days following app advice than those preceding app advice (0.45 versus 0.34; *P*<.001). The positive relation between counselor app advice and app usage 24 hours after receiving advice was stronger among smokers with no plan to quit than in those planning to quit (*P*=.03), independent of education and phone or app problems.

**Conclusions:**

Findings show the utility of supportive accountability for increasing smoking cessation app use in a predominantly low-income, minority population, particularly if quit motivation is low. Results also highlight the importance of addressing personal and phone/technical barriers in addition to adding supportive accountability.

**Trial Registration:**

ClinicalTrials.gov NCT02602288; https://clinicaltrials.gov/ct2/show/NCT02602288

## Introduction

Mobile apps can offer convenient, low-cost, and on-demand support and intervention for smoking cessation. According to estimates, approximately 63%-76% of smokers own a smartphone [1,2], and hundreds of thousands of smokers download cessation apps monthly [3]. Mobile apps could serve as a standalone intervention or an adjunct to other behavioral interventions, such as telephone quitlines. Unfortunately, users’ low engagement with smoking cessation apps makes it difficult to evaluate their effectiveness [4]. In general, app abandonment is problematic: approximately one-fifth of apps are abandoned after one use [5] and over half within a month [6]. Presumably, greater utilization of cessation apps would increase their effectiveness. One study showed that fully adherent users of smoking cessation apps were more than four times as likely to abstain as nonadherent users, but only 24% were fully adherent [7]. This study aims to identify factors that increase smoking cessation app use to inform theory and future interventions.

The investigation was guided by the supportive accountability model, which maintains that adherence to eHealth interventions, including mobile health apps, can be increased through accountability to a supportive, trustworthy person with relevant expertise, such as a health coach or medical provider [8]. The model aligns with prior health behavior research and theory on treatment adherence. For example, social support from interventionists is positively related to treatment adherence across various medical treatments and health-related behaviors [9]. In one smoking cessation treatment study, social support was associated with higher nicotine patch adherence [10]. Clinical practice guidelines also underscore the importance of intratreatment support in professionally delivered cessation interventions [11]. The quality of support is important. Accountability born out of a drive to please a respected coach or health care provider is likely to be more effective than accountability born out of duress (eg, shame, fear, perceived penalties) [12].

Drawing upon self-determination theory [13], the supportive accountability model predicts that motivation to change a behavior can moderate the effect of supportive accountability on health behavior change [8]. Specifically, the more intrinsically motivated a person is to change a behavior, the less social support (ie, extrinsic motivation) they may require. Intrinsic motivation, which is reflected in behavior change intentions [14], has been linked to behavior change efforts and success. For example, higher intention to quit smoking has been linked positively to smoking abstinence [15], quit attempts, and use of electronic nicotine devices to reduce smoking [16]. Another corollary based on self-determination theory is that supportive accountability will become less necessary as individuals progress from being extrinsically motivated to being internally motivated to reach their goals [8]. Indeed, under these latter conditions, ongoing supportive messaging could be construed as controlling or signal that the support provider doubts the support recipient’s ability.

This observational study investigates the relations between supportive accountability, motivation, and smoking cessation app use in the context of a clinical trial aimed to promote smoking cessation among low-income maternal smokers. The trial, Babies Living Safe and Smokefree (BLiSS) [17], targeted mothers who smoke and live in predominantly low-income and minority neighborhoods in a major US city. This population was targeted because children in these communities have an excess burden of environmental tobacco smoke exposure (TSE) [18]. We were especially interested in evaluating the uptake and usage of a mobile smoking cessation app in this population because compared to non-Hispanic White smokers, non-Hispanic Black and Hispanic smokers are less likely to use tobacco-cessation aids during a quit attempt [19]. Identifying correlates of app utilization in this high-risk population could inform future smoking cessation interventions that incorporate mobile apps. Further, this analysis provides a theoretical test of the supportive accountability model in an understudied population.

We tested two hypotheses based on the supportive accountability model:

1. A higher percentage of smokers will use a cessation app on their phone in the 24 hours after receiving prompts about app usage from a cessation counselor (ie, supportive accountability) than in the 24 hours preceding such prompts.
2. The relation between prompting and app usage will be stronger among participants not planning to quit in the next three months than among participants planning to quit in the next three months (ie, motivation as a moderator).

We also explored correlates and potential barriers to app usage. As the target population is low income, we anticipated some potential technical and phone-related barriers (eg, service disruptions due to late payments, phone sharing) as well as practical barriers (eg, no time, lack of interest). Finally, we explored whether app usage correlated with amount of smoking at end of treatment.

## Methods

### Study Overview

This investigation used secondary data collected as part of the BLiSS trial [17]. BLiSS used a randomized two-group design with three measurement points: baseline, 3-month follow-up, and 12-month follow-up. Outcomes include bioverified child TSE and bioverified maternal quit status. Maternal smokers with children <6 years old were recruited from government-subsidized clinics that deliver the Special Supplemental Nutrition Program for Women, Infants, and Children (WIC). All study participants received a WIC system-level intervention based on the Ask, Advise, Refer (AAR) clinical best practice guidelines established by the American Academy of Pediatrics [20]. WIC nutrition counselors delivered AAR. After a WIC referral, the trial's project manager randomized eligible and consented mothers to either a 3-month multimodal behavioral intervention (AAR + MBI) targeting parental smoking, or a 3-month attention control intervention (AAR + Control) targeting family nutrition. The appropriate Institutional Review Board approved all study procedures, and all participants provided informed consent to participate. This observational study is limited to the participants in the AAR + MBI arm of the BLiSS trial and app usage patterns and correlates, not trial outcomes.

### Participants

Trial eligibility criteria included the following: received WIC clinic AAR intervention; English-speaking; at least 18 years old; report smoking; own a smartphone; and report their child aged <6 years is exposed to tobacco smoke. Exclusion criteria included the following: currently pregnant; presenting issues that could interfere with their ability to provide informed consent or follow study procedures, such as psychosis, inadequate health literacy, or non-nicotine drug dependence. All BLiSS participants randomized to the AAR + MBI treatment arm (N=199) were potentially eligible for inclusion in this observation study. However, 18 participants were excluded from all analyses, leaving a sample of 181. Reasons for exclusion included technical problems downloading the mobile app (n=6), issues with the back-end software that tracked participants' app usage (n=6), and participant withdrawal from the trial before receiving intervention or app advice (n=6). Comparisons of excluded and nonexcluded participants revealed no statistically significant differences in age, race, marital status, employment status, education level, or phone operating system.

### Procedures

After WIC staff referred mothers to the trial, trained research assistants screened for eligibility, administered informed consent, and collected baseline self-report data using computer-assisted telephone interviews. Participants were then randomized to either the AAR + MBI or AAR + Control condition. The AAR + MBI intervention included messaging about child TSE harms as well as support and guidance with skills training and problem-solving delivered via multiple channels: the project quitline, providing up to 5 telephone counseling sessions over 3 months; cessation mobile app; print materials for the participant and their family; intersession text follow-up, reminders, and support, as well as educational video clips that reinforced telephone session and written materials content; and 8 weeks of nicotine replacement therapy and instructional support.

After randomization, participants in the AAR + MBI condition had an orientation home visit that provided a review of intervention objectives, a binder of intervention print materials, and an illustrated guide to using the mobile app. Research staff also assisted participants in downloading the mobile app and showed them a brief video tutorial on how to use the app.

Telephone counselors delivering the skills training and support intervention received intensive training in tobacco treatment enhanced with support, advice, and problem solving around protecting children from tobacco smoke exposure and creating a smoke-free home and car. Importantly, they were trained in how to use a telephone counseling process that would promote participants' mobile app usage to complement and extend treatment beyond the phone sessions. For example, telephone counseling included guidance on goal setting, building social support, and improving skills (eg, self-monitoring) to reduce child TSE, identify smoking triggers, and manage urges to smoke. The mobile app has tools that support all these processes.

**Figure 1:**
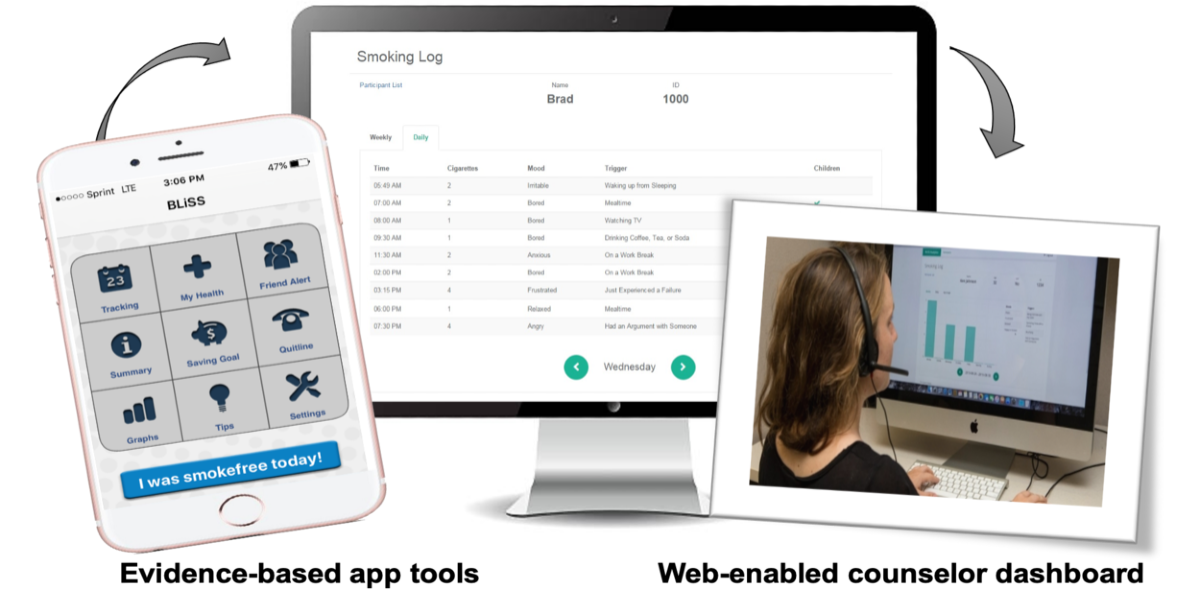
QuitPal-m app features and illustration of web-enabled counselor dashboard.

The BLiSS mobile app was a modified version of the National Cancer Institute's QuitPal app [21]. The content and tools provided by the QuitPal app are grounded in evidence-based research and US clinical practice guidelines for treating tobacco use and dependence, which makes it stand out among smoking cessation apps [3,22]. Originally designed as a standalone intervention for iOS-based phones, the modified QuitPal app (QuitPal-m) works on both iOS and Android platforms. Key features are shown in Figure 1. QuitPal-m includes features that promote goal setting (eg, quit date, financial goals), real-time self-monitoring of number of cigarettes smoked and cigarettes smoked with children in the same room, and monitoring of mood and context associated with smoking episodes. The app has algorithms that use tracking data and goals to send personalized notifications with tips about smoking triggers and managing cravings, as well as motivational reminders that coincide with progress (eg, health milestones and money saved by reducing or quitting smoking). Other features include goal progress summary and connectivity to the BLiSS quitline and to social media to alert friends of progress and build support for quitting. A video recording tool from the original QuitPal app was excluded from QuitPal-m to facilitate ease of use and to emphasize content and processes covered in the telephone counseling sessions.

An innovative feature of QuitPal-m is a web-linked portal that connects telephone counselors to a dashboard that displays participant app usage (Figure 1). Counselors were trained to review dashboard data before counseling phone sessions to guide their supportive feedback about app usage and the behavior change progress during phone sessions. Counselors aimed to drive participants' early adoption of tracking and responding to app reminders. Counselors also could provide positive reinforcement about tracking efforts and progress, review behavioral patterns emerging over time, and offer to troubleshoot challenges to app usage and behavior change efforts with nonusers. For those participants who readily engaged with the self-monitoring functions of the app, counselors could shift their attention to suggesting how the app could be used to address specific cessation challenges raised during counseling calls. Typically, app advice was provided in each counseling phone session, unless time was limited and other topics took precedence based on a participant’s progress. For example, if a participant missed a phone session, a counselor might have to cover topics from two sessions and not have a chance to address app usage. Counselors' session notes included a field for recording if app usage was discussed as part of the counseling session.

During the home visit orientation to the app, participants were told that the smoking self-monitoring, or tracking, features were the most important and should be used daily. Home visitors demonstrated how to enter data into the app. Participants also were informed about the counselor dashboard and how telephone counselors would routinely monitor app entries to learn more about participants' smoking habits and guide their advice about the participants' behavior change efforts. Finally, they were told that the counselor would remind them or initiate troubleshooting when app use adherence was low. Thus, participants were aware of the expectations, monitoring, and accountability related to app usage from the beginning of the intervention.

### Measures

#### QuitPal-m App Adherence

Adherence is defined as the active use of the app as recommended [23]. In this study, BLiSS AAR + MBI participants were encouraged to use the app daily. Thus, the primary outcome of interest was the number of days the app was used (range 0 to 91). Each participant's phone was linked to a back-end software program that recorded the days when the app was launched, as well as specific features that were accessed and any inputted data. To count as use, a participant had to launch the app and use one of the eight features (eg, input smoking data, view savings, request a tip).

#### Adherence to Usage Advice

Another important outcome to test the supportive accountability model was the proportion of days that participants used the app 24 hours before and 24 hours after receiving advice from their counselor to do so. This usage was calculated by dividing the total number of days the app was used 24 hours after (or before) advice was offered divided by the total number of days advice was offered. For example, if a person received advice on three days and used the app within 24 hours on each of those three days, they would score 100 (3/3). If they only used the app within 24 hours on two of the days, they would score 66 (2/3). Thus, scores could range from 0 to 100.

#### App Helpfulness and Barriers to Use

Participants rated the app helpfulness on a 4-point scale (1=not at all, 2=a little helpful, 3=somewhat helpful, 4=very helpful) postintervention. They also reported (no/yes) whether they experienced any of the following barriers to app usage during the intervention: forgetting, lack of interest, lack of time, and confusion/difficulty using the app. In addition, we tracked phone and technical problems reported throughout the study that interfered with app use (eg, phone not in service, app freezing).

#### Motivation and Smoking Behavior

To assess participants' motivation/determination to quit smoking, we included a baseline question about whether they planned to quit smoking in the next 3 months (no/yes). We also included a self-report measure of average cigarettes smoked per day in the past week at the 3-month end-of-treatment period.

## Results

The sample (N=181) was comprised of mothers who were mostly single (113/181, 62.4%), Black (123/181, 68.0%), and unemployed (105/181, 58.0%). The highest level of education completed for the majority was high school or less (111/181, 61.3%). The most common phone operating system was Android (144/181, 79.6%), followed by iOS (37/181, 20.4%). At baseline, over three-fourths (138/181, 76.2%) of the participants reported that they were planning to quit smoking in the next three months.

**Figure 2:**
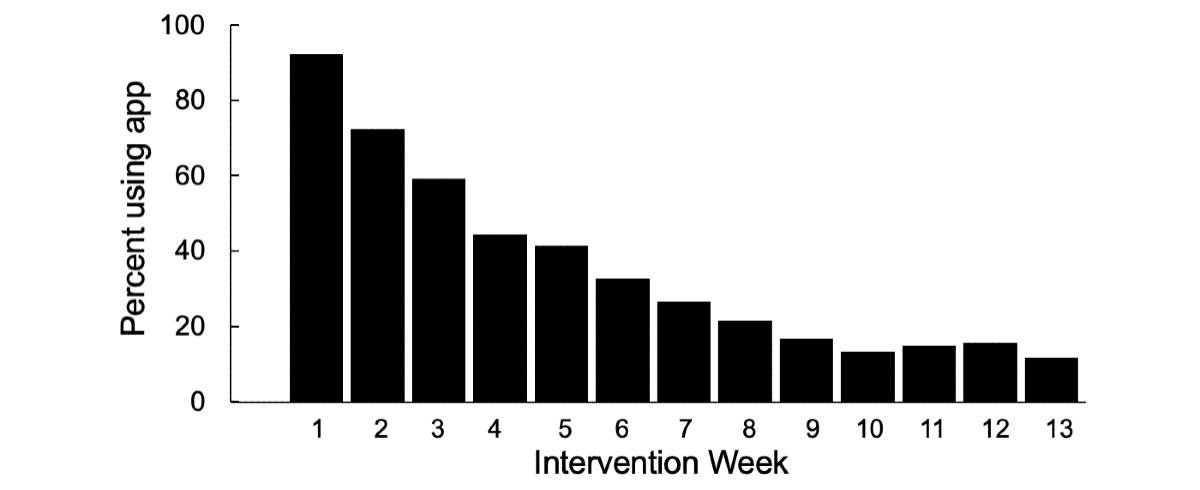
Percent of participants using QuitPal-m app by intervention week (N=181).

On average, participants received advice to use the app 3 times (median 3; mean 2.98, SD 1.58) over the course of the intervention. A total of 10 of the 181 participants (5.5%) received no advice: 9 because they could not be reached for phone intervention sessions and 1 because the interventionist did not have an opportunity to bring it up during the single phone session the participant completed. Patterns of app usage are shown in Table 1. The most frequently used feature was the tracking of cigarettes smoked, with all other features used rarely. As shown in Figure 2, app usage was greatest during the first week of treatment and declined rapidly over time. On average, participants used the app on 16 days over the entire intervention period, and fewer than 50% (80/181) used the app after week 4. No participants used the app daily, as recommended, although one person used the app 87/91 days.

**Table 1:** Patterns of app usage (N=181).

Variable	Mean (SD)	Range
Days app used (out of 91)	16.48 (17.52)	0-87
Times tracking feature used	63.64 (81.68)	0-443
Times savings feature used	1.72 (3.09)	0-26
Times graph feature used	1.36 (2.43)	0-16
Times tips feature used	0.92 (1.85)	0-16
Times summary feature used	1.23 (2.46)	0-15
Times friend alert feature used	0.87 (1.69)	0-14
Times my health feature used	1.48 (2.17)	0-12
Times quitline phone number used	0.39 (0.87)	0-14

Examining the relation of days of app usage to sociodemographic factors revealed a single significant association, with education: participants used the app on significantly more days if they had more than a high school education (mean 21.49, SD 21.37) than if their highest education was high school or less (mean 13.32, SD 13.77; *t*_179_=3.13, *P*=.002). Frequency of app usage was unrelated to participants' age, number of years smoking, level of dependence on cigarettes, marital/partnered status, and employment status. Consistent with the study’s premise that greater adherence to app use can improve outcomes, more days of app use was negatively correlated with number of cigarettes smoked per day at the end of treatment (*r*=–.25, *P*<.001).

Of the 155 participants who completed the postintervention survey, most rated the app as very helpful (103/155, 66.5%) or somewhat helpful (26/155, 16.8%), a minority (23/155, 14.8%) rated it as not at all/a little helpful, and a few did not answer (3/155, 2%). Participants who rated the app as very helpful tended to open the app on significantly more days (mean 20.56, SD 19.44) than their counterparts who rated it less helpful (mean 13.56, SD 13.26; *t*_153_=2.34, *P*=.02). A large minority (71/171, 41.5%) reported experiencing barriers to app usage during the intervention. Ordered from most to least common, barriers included the following: forgetfulness (28/171, 16.4%), lack of time (28/171, 16.4%), lack of interest (26/171, 15.2%), and confusing/difficult to use (26/171, 2.3%). Participants who reported any barrier during the intervention opened the app on significantly fewer days (mean 12.51, SD 14.08) than their counterparts who reported no barriers (mean 20.58, SD 19.29; *t*_169_=3.00, *P*=.003).

A sizeable proportion (68/181, 37.6%) of the participants reported phone and other technical problems that occasionally interfered with using the app at some time during the intervention. This is in addition to the 12 who were excluded from the study due to app download and back-end issues from the start. The most frequently reported problem was intermittent service disruptions due to running out of minutes or being unable to pay bills on time (33/181, 18.2%), followed by deleting the app due to software problems (eg, app freezing) or insufficient memory for the app (23/181, 12.7%), followed by getting a new phone and having difficulty downloading the app again (12/181, 6.6%). Certain problems, such as phone service disruption, affect some app features and functions, such as communication between the app and the counselor dashboard, but not other features, such as tracking, savings, and tips. Participants who experienced phone/technical problems used the app on significantly fewer days (mean 11.40, SD 13.04) than their counterparts who did not experience these problems (mean 21.49, SD 19.86; *t*_179_=4.04, *P*<.001).

Finally, we analyzed patterns of usage following advice from the counselor and whether motivation/determination to quit moderated the effects of counselor advice on app usage. These analyses excluded the 10 participants who received no advice. Paired *t* tests showed that, on average, participants used the app a greater proportion of the days following app advice (mean 0.45, SD .37) than days preceding app advice (mean 0.34, SD .35; *t*_170_=4.35, *P*<.001). To investigate if supportive accountability increased app usage, particularly among those who were not planning to quit (low motivation), we used multiple regression. The outcome was proportion of days using the app within 24 hours after receiving counselor advice. The predictors included total number of times advice was given, plan to quit in next three months (yes/no), and the interaction between amount of advice and plan to quit. Variables were centered around zero prior to creating the cross-products for the interaction term. Other covariates included factors known to predict the number of days participants used the app: education, app ever unavailable due to phone/technical problems, and perceived barriers to app usage.

**Figure 3:**
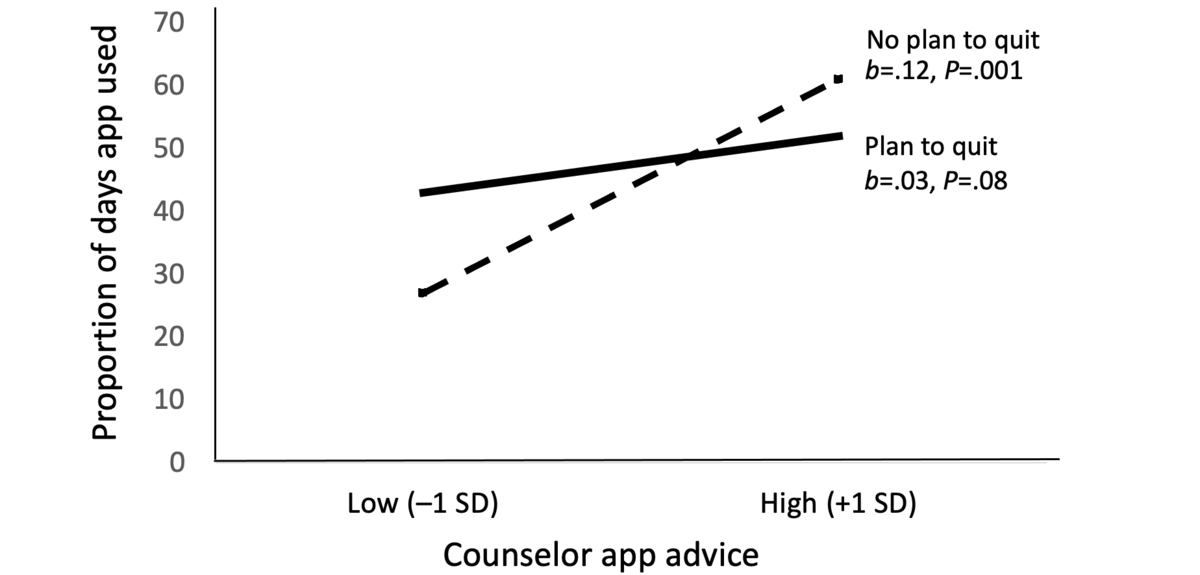
Relation between counselor app advice and proportion of days app was used within 24 hours after advice as a function of motivation to quit smoking (N=171).

As shown in Table 2, there was a significant main effect of advice, no main effect of planning to quit, and a significant advice × planning to quit interaction on likelihood of app usage within 24 hours after receiving advice. Figure 3 plots the interaction. Simple effects reveal that counselor advice was positively and significantly related to more app usage among those participants who were not planning to quit at the beginning of the study (ie, they were least motivated to change behavior). The simple slope relating advice to app usage was positive but not statistically significant among those who were planning to quit at the beginning of the study.

**Table 2:** Regression models predicting proportion of days app was used within 24 hours after receiving app advice from a counselor (N=171).

Predictor	*B* (SE)	*T* value	*P* value
Constant	.56 (.05)	12.10	<.001
Highest education level^a^	.06 (.05)	1.12	.27
App ever unavailable due to phone/technical problems^b^	–.16 (.06)	–2.82	.005
Reported any barriers to app use^b^	–.16 (.05)	–3.00	.003
Total counselor advice to use app	.05 (.02)	3.28	.001
Plan to quit in next 3 months^b^	.05 (.06)	.79	.43
Total advice × plan to quit	–.08 (.04)	–2.25	.03

^a^0=high school or less, 1=more than high school.

^b^0=no, 1=yes.

## Discussion

### Principal Results

Overall adherence to daily app use goals was low and declined steadily over the course of the intervention. Consistent with the supportive accountability model [8], counselor monitoring and supportive advice about app usage was positively associated with app usage. Also consistent with the model, accountability to a counselor appeared to increase app engagement more among smokers who were not planning to quit than among their counterparts who were already planning to quit. Thus, adding supportive accountability to tobacco cessation interventions that deploy mobile apps can improve adherence, which we hypothesize would enhance intervention effectiveness in research or practice contexts. The observed negative correlation between days of app use and cigarettes smoked per day at the end of treatment is consistent with this expectation. Personal and nuisance factors also were linked to lower app use, including lower educational achievement, perceived barriers (eg, forgetfulness, no time or interest), and phone/technical problems. Altogether, these findings suggest that accountability to a trusted, supportive expert can increase adherence to mobile app treatment elements, but other factors also play a role.

The current findings suggest that supportive accountability is a promising method to improve adherence to mobile app use, especially among users with low levels of motivation to change. The interaction between accountability and motivation suggests that increased motivation is a primary mechanism of supportive accountability in app adherence. Intervention models, such as BLiSS, that incorporate mobile apps and interaction with counselors can build accountability directly into the counseling protocol. Folding in supportive accountability processes and messaging into treatment-goal setting and skills-training elements of behavioral interventions can maximize the chance that participants benefit as much as possible from app engagement. For example, getting participants to engage in self-monitoring/tracking as “homework” and taking stock of their smoking patterns, including triggers and consequences of smoking that sustain the behavior, may boost intervention efficacy. Emphasizing accountability messaging with nonadherent users could have the twin virtues of supporting users with the greatest need and of not alienating users who are already highly engaged.

A final noteworthy finding was the relatively high participant ratings of the helpfulness of the app. Further, the more satisfied participants were with the app, the more they tended to use it. Thus, increasing user satisfaction could help to promote more engagement. These findings appear to conflict with the overall low engagement and rapid drop-off in app use during the intervention. It is possible that participants were able to extract the primary value from the app in less time than we originally predicted. In retrospect, we realize that it would not take more than a week or two of tracking for users and counselors to recognize smoking behavior patterns and, on that basis, make smoking avoidance/cessation strategies. In addition, after several days of using the tracking feature, the app generates automated notifications about health improvements and personalized tips for avoiding smoking without the user having to launch the app daily and manually input data. Apps that use other strategies to address smoking, such as stress management, may be helpful over longer periods of time when relapse prevention is important. However, for initial quit attempts, when it is important to identify and address factors that trigger and sustain smoking, an app with a more circumscribed time of use may be sufficient.

An interesting question for future research is whether the medium and source of supportive accountability influences adherence levels. For example, is an SMS text message from a counselor as effective as direct contact and collaborative problem solving that can occur during a live counseling session? Is a supportive accountability approach as effective at improving app adherence when it is automated or delivered by an embodied conversational agent (eg, a computer-generated avatar of a counselor) versus a live human? Unlike common reminder software, embodied agents can be verbally expressive and mimic human gestures, which could elicit social responses from people that parallel human-human social interaction. If effective, such an approach would reduce some of the burden on the interventionist.

A related question is how much encouragement or nudging from a coach is beneficial for promoting app usage? The supportive accountability model suggests that once an individual has internal motivation to change a behavior, ongoing accounting may backfire, or at least show diminished returns. In this study, the amount of direct advice from the health counselor was deliberately modest and it was front-loaded to the early weeks of the intervention. However, as we did not experimentally manipulate frequency and intensity of supportive accountability-driven app advice and feedback, it raises the question of whether more advice might have resulted in greater adherence.

In thinking broadly about the challenges of app engagement, it is important to consider the social and economic contexts that influence user engagement. In this study, participants were drawn from predominantly minority and low-income communities. As reflected in some of the observed barriers (eg, phone service disruptions, phone sharing, time constraints), participants' life circumstances would have undermined adherence even among those motivated and otherwise engaged in the overarching multimodal intervention. Linking the QuitPal-m app to wearable smoking sensors [24] might overcome some contextual factors (eg, time constraints) that disrupt app usage and reduce the burden of tracking smoking. The introduction and eventual widespread availability of 5G will potentially overcome other barriers, such as app connectivity problems. Another noted barrier was lack of interest, which might be improved by amplifying some of the game-like elements of QuitPal-m, such as social connectivity, financial savings graphs, and praise for achieving goals [25].

### Limitations

This study has some noteworthy limitations. The primary one is the lack of experimental data. Instead of manipulating levels of supportive accountability, we measured it and observed how it related to app use behaviors. This correlational design is subject to internal validity threats, including the possibility that something other than accountability increased participants' app use after receiving counselor advice. For example, maybe the call itself served as a reminder and did not require the counselor to specifically discuss the app. Future research will need to add randomization and control conditions to rule out alternative explanations of findings. Another limitation is that participants used their own cell phones and service plans. This introduced extraneous factors that interfered with app use independent of users' intentions/desires to use the app. A controlled study would be able to isolate the effects of supportive accountability by providing a device and service, ensuring equitable access for all participants.

### Conclusions

The findings show the potential utility of supportive accountability for increasing use of a smoking cessation app in a low-income, predominantly minority population. Consistent with the model advanced by Mohr and colleagues [8], supportive accountability-driven app advice was most helpful for smokers with low motivation to change their smoking behavior. This finding suggests that it might be possible to target messaging based on individuals' stage of change, or progress in treatment (eg, preparing to quit, initial quit phase, or efforts to maintain longer-term abstinence). Finally, we found that participants' social and economic life contexts influenced app use. Addressing these factors, including time constraints, interest level, and access to affordable high-quality phones/devices and service will also help improve app use.

## Abbreviations

AAR: Ask, Advise, Refer
BLiSS: Babies Living Safe and Smokefree
MBI: multimodal behavioral intervention
QuitPal-m: modified QuitPal app
TSE: tobacco smoke exposure
WIC: Special Supplemental Nutrition Program for Women, Infants, and Children
